# A Systematic Review of Antibiotic Resistance Trends and Treatment Options for Hospital-Acquired Multidrug-Resistant Infections

**DOI:** 10.7759/cureus.29956

**Published:** 2022-10-05

**Authors:** Walter Y Agyeman, Aakash Bisht, Ankit Gopinath, Ameer Haider Cheema, Keyur Chaludiya, Maham Khalid, Marcellina Nwosu, Srujana Konka, Safeera Khan

**Affiliations:** 1 Internal Medicine, Piedmont Athens Regional Medical Center, Georgia, USA; 2 Internal Medicine, California Institute of Behavioral Neurosciences & Psychology, Fairfield, USA; 3 Internal Medicine, Government Medical College, Amritsar, Amritsar, IND; 4 Internal Medicine, Kasturba Medical College, Manipal, Manipal, IND

**Keywords:** hospital-acquired infections, multidrug resistance, antibiotic treatment, extensive drug resistance, pan drug resistance, antimicrobial resistance, antibiotic resistance

## Abstract

Antimicrobial resistance is a major public health challenge described by the World Health Organization as one of the top 10 public health challenges worldwide. Drug-resistant microbes contribute significantly to morbidity and mortality in the hospital, especially in the critical care unit. The primary etiology of increasing antibiotic resistance is inappropriate and excessive use of antibiotics. The alarming rise of drug-resistant microbes worldwide threatens to erode our ability to treat infections with our current armamentarium of antibiotics.

Unfortunately, the pace of development of new antibiotics by the pharmaceutical industry has not kept up with rising resistance to expand our options to treat microbial infections. The costs of antibiotic resistance include death and disability, extended hospital stays due to prolonged sickness, need for expensive therapies, rising healthcare expenditure, reduced productivity from time out of the workforce, and rising penury. This review sums up the common mechanisms, trends, and treatment options for hospital-acquired multidrug-resistant microbes.

## Introduction and background

Hospital-acquired multidrug-resistant microbes are a significant cause of morbidity and mortality, especially in the critical care unit. It is a significant public health threat that prolongs hospital stays and increases healthcare costs. In 2019, an estimated 4.95 million deaths were associated with bacterial antimicrobial resistance alone [1]. Approximately, 1.2 million deaths per year are directly attributable to bacterial antimicrobial resistance alone [1,2].

Antibiotic resistance is classified into three broad groups according to the sensitivity pattern to the different antibiotic classes, namely, pan-drug-resistant (PDR), extensively drug-resistant (XDR), and multidrug-resistant (MDR) microbial infections. MDR microbes are resistant to at least one agent from three or more antibiotic classes. XDR microbes are resistant to at least one agent from each antibiotic class except two or fewer classes. Lastly, PDR microbes are resistant to an agent from all antibiotic classes [3].

The rising incidence of MDR microbes is a safety concern for patients, clinicians, and healthcare administrators. Risk factors for acquiring MDR infections are associated with medical treatment and healthcare facilities, i.e., recent antibiotic use (<90 days), catheter or medical device carriage, and prolonged stay in a healthcare facility [4]. Hospital-acquired or nosocomial infections occur at least 48 hours after admission in a healthcare delivery setting, including hospitals and long-term care facilities. They may also arise after discharge from a healthcare facility [5]. Nosocomial infections put patients and healthcare staff at risk. Long-term care facilities are a proposed connecting link in spreading MDR infections between the hospital and the community [6].

The emergence of antimicrobial resistance is a result of the indiscriminate use of antibiotics in the healthcare, veterinary, and agricultural industries. Wrong antibiotic choice, inadequate dosing, and unnecessarily extended treatment drive antibiotic resistance within hospitals and other healthcare settings, such as nursing homes and the community. Much work has been done to describe antimicrobial resistance genes. However, we need to interrogate the trends in antimicrobial resistance and treatment options for MDR infections to inform and guide public health policy, antimicrobial stewardship programs, and clinical treatment guidelines. This systematic review describes recent antibiotic resistance trends and treatment options for hospital-acquired MDR infections.

## Review

Reporting guideline

This systematic review was written according to the Preferred Reporting Items for Systematic Reviews and Meta-Analyses (PRISMA) 2020 guidelines [7].

Databases and search strategy

A systematic search was conducted using PubMed, PubMed Central (PMC), ScienceDirect, and Cochrane Library register for all databases on January 16, 2022. The field search was done on PubMed using Medical Subject Headings (MeSH) and keywords. We searched the other databases using the keywords hospital-acquired infection, cross-infection, microbial drug resistance, and treatment. PubMed Search Builders were created using the Boolean scheme, as shown in Table 1.

**Table 1:** The bibliographic search strategy.

Concept	Keywords	PubMed Search Builder
Hospital-acquired infection	Hospital-acquired infection, cross-infection	(“Cross Infection/drug therapy”[MeSH] OR “Cross Infection/etiology”[MeSH] OR “Cross Infection/microbiology”[MeSH] OR “Cross Infection/prevention and control”[MeSH] OR “Cross Infection/therapy”[MeSH] OR “Cross Infection/transmission”[MeSH])
Microbial drug resistance		(“Drug Resistance, Microbial/analysis”[MeSH] OR “Drug Resistance, Microbial/drug effects”[MeSH] OR “Drug Resistance, Microbial/epidemiology”[MeSH] OR “Drug Resistance, Microbial/etiology”[MeSH] OR “Drug Resistance, Microbial/prevention and control”[MeSH] OR “Drug Resistance, Microbial/statistics and numerical data”[MeSH] OR “Drug Resistance, Microbial/therapy”[MeSH] OR “Drug Resistance, Microbial/trends”[MeSH])

We pooled the keywords using the Boolean term “OR” and combined their corresponding search builders that we obtained from PubMed using MeSH terms. In addition, we applied restrictions to MeSH-major topics. All concepts and keywords were combined into a final search strategy using the Boolean term “AND,” as shown in Table 2.

**Table 2:** The MeSH strategy and corresponding filters.

Full MeSH strategy	Number of articles
(“Cross Infection/drug therapy”[MeSH] OR “Cross Infection/etiology”[MeSH] OR “Cross Infection/microbiology”[MeSH] OR “Cross Infection/prevention and control”[MeSH] OR “Cross Infection/therapy”[MeSH] OR “Cross Infection/transmission”[MeSH]) AND (“Drug Resistance, Microbial/analysis”[MeSH] OR “Drug Resistance, Microbial/drug effects”[MeSH] OR “Drug Resistance, Microbial/epidemiology”[MeSH] OR “Drug Resistance, Microbial/etiology”[MeSH] OR “Drug Resistance, Microbial/prevention and control”[MeSH] OR “Drug Resistance, Microbial/statistics and numerical data”[MeSH] OR “Drug Resistance, Microbial/therapy”[MeSH] OR “Drug Resistance, Microbial/trends”[MeSH])	2,895 articles obtained after applying filters (filters: articles published in the last five years, articles published in the English language, patients older than 12)

Inclusion and exclusion criteria

The population of interest includes patients admitted to the hospital for at least 48 hours with a culture or antigen/polymerase chain reaction (PCR)-confirmed microbial diagnosis. Our intervention is any novel antibiotic treatment. The comparator is the standard of care antibiotic treatment, and the outcome of interest is recovery as defined by clinical and microbiological cure-culture negative after antibiotic treatment.

The literature search was conducted to identify relevant studies that examine antibiotic resistance trends and treatment for hospital-acquired MDR infections. Inclusion criteria were studies conducted on the adult population and published in English as full-text papers in the past five years. Studies in the pediatric population, unpublished literature, papers older than 2016, irrelevant, non-full-text, gray, case reports, editorials, and non-English reports were excluded.

Screening of articles

After obtaining the relevant articles from the databases, we removed duplicates using Microsoft Excel. We subsequently screened the articles based on title, abstract, and reading full-text articles. Articles were screened based on their likelihood of yielding clinically significant practice changes as determined by the writing committee. Finally, we subjected all short-listed articles to a quality appraisal.

Quality appraisal

As displayed in Table 3, we assessed the short-listed articles for quality and risk of bias using tools depending on the study type. Each assessment tool had its criteria and scoring. A score of at least 60% for each assessment tool was accepted.

**Table 3:** Quality appraisal tools used to assess the various types of studies.

Type of study	Quality appraisal tool
Narrative reviews	Scale for the Assessment of Narrative Review Articles 2 (SANRA 2)
Observational studies	Newcastle-Ottawa Scale
Randomized controlled trials	Cochrane Collaboration Risk of Bias Tool
Systematic reviews and meta-analyses	Assessing the Methodological Quality of Systematic Reviews (AMSTAR 2)

**Figure 1:**
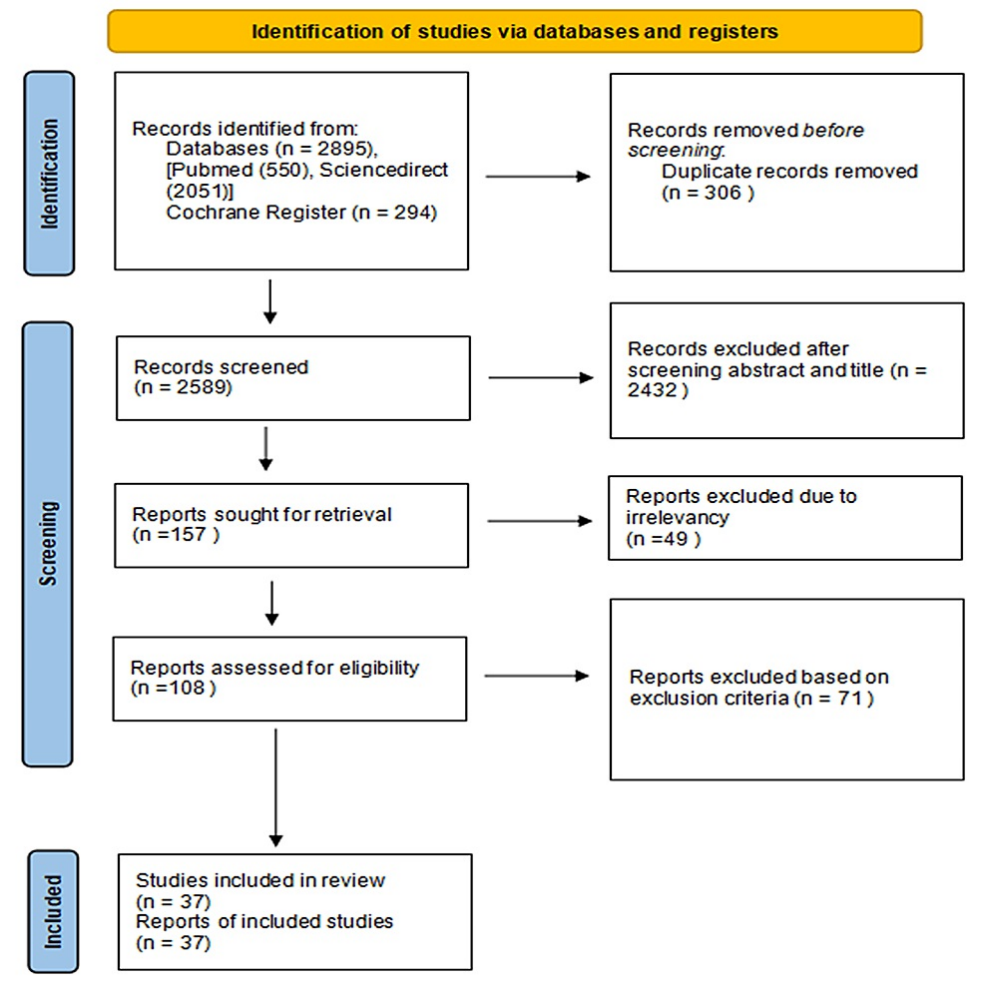
The PRISMA flow diagram of the study search of databases and registers. PRISMA: Preferred Reporting Items for Systematic Reviews and Meta-Analyses

A total of 2,895 articles were found upon employing the appropriate keywords. A total of 306 duplicates were filtered out before screening; 2,589 articles underwent the screening process, of which 2,432 articles were removed based on their titles and abstracts. The authors retrieved 157 articles to assess the full text for relevancy and screened 108 reports for eligibility. In total, 37 articles were finally included in the review upon an in-depth analysis of quality, inclusion/exclusion criteria, and study designs. The first two authors conducted the data extraction and appraised the studies independent of each other. Whenever there arose a difference of opinion, the writing committee settled the outcome. The search strategy and the process of selecting the final studies included in this review are depicted in Figure 1.

Because of the variability, such as heterogeneity of participants, interventions, and outcome measures, between studies, this systematic review describes these trials and reviews based on their outcomes, applicability, and limitations on a narrative synthesis rather than conducting a meta-analysis.

Two independent investigators (the first and second author) performed article selection, assessment, and analyses in each step. If there was a contradictory result regarding an article’s eligibility, its full text was assessed by consensus within the group.

We evaluated randomized controlled trials (RCTs) in this study using the Cochrane Collaboration risk of bias tool. Seven RCTs were reviewed. Four were included, and three were rejected due to at least one substantial risk of bias in any domain. The results of the quality assessment are shown in Table 4.

**Table 4:** Quality Assessment using the Cochrane Collaboration risk of bias tool.

Article	Random sequence generation	Allocation concealment	Double blinding	Attrition bias	Reporting bias	Blinding outcome assessment	Other bias
Khorvash et al. [8]	Low	Low	Low	Low	Unclear	Unclear	Low
Harris et al. [9]	Low	Low	High	Low	Low	Unclear	Low
Arthur et al. [10]	Low	Low	Low	Low	Unclear	Low	Low
Salomão et al. [11]	Low	Low	Low	Low	Unclear	Unclear	Low
Davey et al. [12]	Low	Low	Low	Unclear	Low	Low	Low
Amin et al. [13]	Low	Low	Low	Low	Unclear	Low	High
Maxwell et al. [14]	Low	High	High	Low	Low	Unclear	Low

We evaluated six systematic reviews using the Assessing the Methodological Quality of Systematic Reviews 2 (AMSTAR 2) criteria., We utilized a passing score of 60% as our cut-off for acceptance. Three articles were included, and three were rejected. The results are displayed in Table 5.

**Table 5:** Quality assessment using AMSTAR criteria for evaluation of selected systematic review studies. AMSTAR: Assessing the Methodological Quality of Systematic Reviews

AMSTAR 2 criteria	Spivak & Hanson [15]	Septimus [16]	Miller et al. [17]	Seo & Song [18]	Effah et al. [19]	Eljaaly et al. [20]
Did the research questions and inclusion criteria for the review include the components of PICO?	No	No	No	Yes	No	Yes
Was an “a priori” design implemented?	No	No	No	Yes	Yes	Yes
3. Did the review authors explain their selection of the study designs for inclusion in the review?	No	No	No	Unclear	Yes	Yes
4. Did the review authors use a comprehensive literature search strategy?	Yes	Yes	Yes	Yes	Yes	Yes
5. Did the review authors perform study selection in duplicate?	Yes	No	Yes	Yes	Yes	Yes
6. Did the review authors perform data extraction in duplicate?	Yes	No	Yes	Yes	Yes	Yes
7. Did the review authors provide a list of excluded studies and justify the exclusions?	No	No	No	No	No	No
8. Did the review authors describe the studies included in adequate detail?	Partial Yes	Yes	Yes	Yes	Yes	Yes
9. Did the review authors use a satisfactory technique for assessing the risk of bias in individual studies that were included in the review?	No	No	No	Unclear	No	Yes
10. Did the review authors report on the sources of funding for the studies included in the review?	Yes	Yes	Yes	Yes	Yes	Yes
11. If a meta-analysis was performed, did the authors use appropriate methods to statistically combine results?	No meta-analysis conducted	No meta-analysis conducted	No meta-analysis conducted	Yes	Yes	Yes
12. If a meta-analysis was performed, did the review authors assess the potential impact of risk of bias in individual studies on the results of the meta-analysis or other evidence synthesis?	No meta-analysis conducted	No meta-analysis conducted	No meta-analysis conducted	Unclear	No	Yes
13. Did the review authors account for risk of bias in individual studies when interpreting/discussing the results of the review?	No	No	No	Unclear	No	No
14. Did the review authors provide a satisfactory explanation for and discussion of any heterogeneity observed in the results of the review?	Yes	Yes	Yes	Unclear	Yes	Yes
15. If they performed quantitative synthesis, did the review authors carry out an adequate investigation of publication bias (small study bias) and discuss its impact on the results of the review?	No meta-analysis conducted	No meta-analysis conducted	No meta-analysis conducted	Unclear	No	Yes
16. Did the review authors report any potential sources of conflict of interest, including any funding they received for conducting the review?	Yes	Yes	Yes	Yes	Yes	Yes
Total score (out of 16)	6.5	5	7	10	10	14
Overall methodological quality	Moderate	low	Moderate	Moderate	Moderate	High

We also reviewed 47 narrative review articles using the Scale for the Assessment of Narrative Review Articles 2 (SANRA 2). A passing score of 70% was utilized as the cut-off. A total of 26 articles were included while 21 narrative review articles were rejected. The results are summarized in Table 6.

**Table 6:** Quality assessment of narrative reviews using the SANRA criteria. 0 (low standard), 1 (moderate standard), 2 (high standard). SANRA: Scale for the Assessment of Narrative Review Articles

Articles	Justification of importance for readership	Statement of aims/formulation of questions	Description of literature search	Referencing	Scientific reasoning	Appropriate presentation of data	Total
Luyt et al. [21]	2	1	1	1	1	1	7
Lee et al. [22]	2	2	1	1	2	2	10
Fernando al. [23]	2	2	1	1	2	1	9
Xia et al. [24]	1	2	1	2	2	1	9
Wong et al. [25]	2	2	1	2	1	1	9
Martin-Loeches et al. [26]	1	2	1	1	1	1	7
Tandogdu et al. [27]	1	2	2	1	1	1	8
Nasr [28]	1	1	1	2	1	1	7
Forsberg et al. [29]	2	1	1	2	1	2	9
Navon-Venezia et al. [30]	2	1	2	1	2	2	10
Gao et al. [31]	1	1	1	1	2	1	7
Niederman [32]	1	1	1	1	1	1	6
Lyons & Kollef [33]	1	1	1	1	1	2	7
Giuliano et al. [34]	1	2	1	2	1	2	9
Yim et al. [35]	1	2	1	1	1	1	7
Lee et al. [36]	2	1	1	1	2	2	9
Dahiya et al. [37]	1	1	1	2	1	1	7
Lockhart et al. [38]	2	1	1	2	2	2	10
Juan et al. [39]	2	2	1	2	2	1	10
Rao et al. [40]	1	2	1	1	2	2	9
Lima et al. [41]	2	1	2	1	2	1	9
D'Accolti et al. [42]	2	1	1	1	2	2	9
Morley et al. [43]	2	1	1	1	2	2	9
Valenzuela-Valderrama et al. [44]	1	2	1	1	2	1	8
Hemeg [45]	2	1	1	1	2	2	9
Bassetti & Righi [46]	1	1	1	1	2	1	7
Gomez-Simmonds & Uhlemann [47]	2	1	1	1	2	1	8
Lynch et al. [48]	2	1	1	1	1	1	7
Bougnoux et al. [49]	2	2	1	1	1	1	9
Septimus & Schweizer [50]	2	1	1	2	2	1	9
Pulzova et al. [51]	1	1	1	2	1	1	7
Li et al. [52]	1	2	1	1	2	1	8
Kidd et al. [53]	2	2	1	1	1	2	9
MacVane [54]	1	1	1	1	2	1	7
Pettigrew et al. [55]	2	1	2	2	2	1	9
Kollef et al. [56]	2	2	2	1	2	1	10
Geisinger & Isberg [57]	2	2	1	2	2	1	10
Cardozo et al. [58]	2	1	1	1	2	1	8
Ruiz-Garbajosa & Canton [59]	1	2	1	1	2	2	9
Chia et al. [60]	1	2	1	1	2	1	8
Silva-Santana et al. [61]	2	1	1	2	1	1	8
Mea et al. [62]	2	1	1	1	2	2	9
Jamal et al. [63]	1	2	1	2	2	1	9
Zhang et al. [64]	1	1	1	2	2	1	8
Dey et al. [65]	2	1	1	1	2	1	8
Pulingam et al. [66]	2	1	1	1	2	2	9

A total of 10 observational/cohort studies were reviewed using the Ottawa Quality Assessment Scale for Cohort Studies. We selected a passing score of 80% as the cut-off. Four studies were included in our final analysis and six studies were not accepted. The results are displayed in Table 7.

**Table 7:** Newcastle-Ottawa Quality Assessment Scale for Cohort Studies. 0 - 0 star, 1 - 1 star, and 2 - 2 stars according to Newcastle-Ottawa Scale.

Articles	Representativeness	Selection	Ascertainment	Demonstration that outcome of interest was not present at start of study	Comparability of cohorts	Assessment of outcome	Long follow-up	Adequacy of follow-up	Total score
Widerstrom et al [67]	1	1	0	1	0	1	1	1	6
Alhumaid et al. [68]	1	1	1	1	1	0	1	1	7
Zaha et al. [69]	1	1	0	1	1	1	0	0	5
Cantón et al. [70]	1	1	1	1	1	0	1	0	6
Puzniak et al. [71]	1	1	0	1	1	1	1	1	7
Wu et al. [72]	1	1	0	1	1	0	1	1	6
Mehl et al. [73]	1	0	1	1	0	1	1	1	6
Bai et al. [74]	1	1	1	1	1	1	1	0	7
Stagliano et al. [75]	1	1	1	0	1	1	1	1	7
Álvarez-Marín et al. [76]	1	1	0	1	0	1	1	1	6

Discussion

Antibiotic Resistance Trends

The various mechanisms by which bacteria can become resistant to an antimicrobial agent due to the diverse resistance genes possessed by different microbial species are being elucidated [24,66]. Antibiotic therapy boosts the emergence of MDR strains through selection pressure and the transfer of genetic resistance elements. Drug resistance arises via de novo mutations during antibiotic use and horizontal transfer of genes via the acquisition of plasmids, transposons, and transferable genetic elements. These antibiotic resistance genes (ARGs) involve altered target binding sites, increased efflux pump activity, enzyme induction, and reduced porins. Extensive drug resistance and pan-drug resistance arise from accumulating multiple resistance gene elements [8]. The main antibiotic resistance mechanisms include active efflux pumps, beta-lactamases, carbapenemases, vancomycin resistance gene (Van A) ligases, and porin deficiency. Various gene families, such as the Ambler ampicillin hydrolyzing class C (*AmpC*), *TEM-1, SHV-1, *cefotaxime hydrolyzing gene(*CTX-M*), and oxacillin hydrolyzing gene (*OXA*), encode beta-lactamases common in bacteria such as *Escherichia coli, Klebsiella pneumoniae, Acinetobacter baumannii, Pseudomonas aeruginosa*, and *Enterobacter spp*. Staphylococcal cassette chromosome *mecA *(*SCCmecA*) and *Mer7* are transferable genetic elements possessed by *Staphylococcus aureus* strains that encode methicillin resistance.

Enterococci are ubiquitous in the environment and present in the natural gut microbiome. Two clinically significant species are *Enterococcus faecalis* and* Enterococcus faecium*. *Enterococcus faecium* is more responsible for fatal invasive hospital-associated infections. Antimicrobial resistance is prevalent in 80-100% of *E. faecium *isolates compared to at most 16% of *E. faecalis* isolates [36]. Enterococci have obtained high-level β-lactam resistance through modification of the penicillin-binding protein gene, resulting in decreased β-lactam affinity and increased β-lactam tolerance due to upregulation of gene expression.

Glycopeptide-resistant enterococci spp*.* have become clinically significant due to the resistance conferred by gene resistance elements of the *Van* family. Risk factors for vancomycin-resistant enterococci infection include invasive gastrointestinal, pulmonary, and urologic procedures, indwelling medical devices, and exposure to fourth-generation cephalosporins. The vancomycin (*Van*) resistance gene family (*Van A, B, C, D, G, H, and L*) encodes enzymes that lead to decreased affinity at the glycopeptide binding site and substitution of the normal precursors, which end in *D-Ala-D-Ala* amino acid sequence [24,66]. The *Van H* gene, also possessed by vancomycin-resistant *Staphylococcus aureus *(VRSA), encodes a dehydrogenase that converts pyruvate to *D-Lac*. The *Van A* gene encodes a ligase which forms an ester bond between *D-Ala* and *D-Lac.* Vancomycin can only combine with the *D-Ala-D-Ala* binding site but not with the *D-Ala-D-Lac *binding site, thus leading to vancomycin resistance.

*K. pneumoniae*, belonging to the *Enterobacteriaceae* family, naturally inhabits the intestinal microbiome. In one study of MDR *K. pneumoniae* isolates in Eastern and South-Western Europe, 50-60% of isolates were resistant to fluoroquinolones, third-generation cephalosporins, and aminoglycosides [30]. The prevalence rate of resistance to commonly used antibiotics in *K. pneumoniae* was 40-80% of isolates in one study in Asia [19,22]. Colistin resistance was identified in 2.9% of *K. pneumoniae* isolates in Asia [19]. The global spread of hypervirulent *K. pneumoniae *strains with extensive antibiotic resistance is worrying. Half of hypervirulent *K. pneumoniae *infections affect patients who are non-elderly and do not have comorbidities, with a mortality rate of up to 40% [22].

*K. pneumoniae* resistance is driven by the accumulation of antibiotic resistance genes leading to XDR strains harboring a super resistome. A super resistome may encompass combinations of carbapenemase genes with aminoglycoside-modifying enzymes or association of *CTX-M* or New Delhi metalloproteinase(*NDM*) carbapenemases, 16S ribosomal ribonucleic acid (*rRNA*) methylases together with porin deficiency and quinolone resistance chromosomal mutations [22]. *K. pneumoniae* can acquire or transfer mobile genetic elements such as transposons from other gram negatives, including *E. coli* and *Serratia marcescens*. Gene elements belonging to the* NDM*,* VIM*,* IMP-1*,and *KPC* (*K. pneumoniae* carbapenemases) enzyme families encode carbapenemases that have an increased activity giving rise to extended-spectrum beta-lactamase (ESBL) Enterobacteriaceae. These enzymes can hydrolyze extended-spectrum cephalosporins. They confer resistance to commonly used beta-lactam antibiotics such as ceftazidime, ceftriaxone, and cefotaxime. Plasmid-mediated resistance genes of all classes have also been identified in *K. Pneumoniae*. The *armA* gene family encodes for enzymes that prevent aminoglycosides from binding to their 16S rRNA target. Other known plasmids gene-mediated 16S rRNA methylases include the *Rmt* family and *NpmA *gene families.

Chromosomal resistance mechanisms that have evolved against aminoglycosides in *K. pneumoniae* include alterations in *AcrAB-TolC* and *KpnEF* efflux pump systems and loss of porins. For fluoroquinolone resistance, the major mechanism is chromosomal mutations in the quinolone binding targets on DNA gyrase involving the *gyrA-gyrB* subunits and topoisomerase IV involving the *parC-parE* subunits. These mutations are also seen in other gram negatives such as *P. aeruginosa* [57]. Overall, fluoroquinolone resistance rates vary geographically but range from 30% to 40% in many countries [43]. Moreover, the *K. pneumoniae* plasmid encodes the *aac *and *qnr* subfamily of genes chromosomally encoded in other gram negatives such as* Citrobacter spp., Stenotrophomonas maltophilia*, and *S. marcescens* that confer resistance to aminoglycoside, fluoroquinolones, and beta-lactams [19].

Porin-mediated resistance in *P. aeruginosa* occurs through mechanisms that downregulate the transcription of the *oprD* gene leading to the deficiency of porins in the outer membrane resulting in decreased susceptibility. Hydrophilic antibiotics, such as β-lactams, tetracyclines, aminoglycosides, and some fluoroquinolones, have been shown to traverse the outer membrane via porins. On the other hand, a decrease in intracellular antibiotic concentration can occur via extrusion through efflux pumps on the membrane. Efflux pumps are classified into six superfamilies. The superfamilies contain (a) the ATP-binding cassette (ABC) superfamily, (b) the small multidrug resistance (SMDR) superfamily, (c) the major facilitator (MF) superfamily, (d) the resistance-nodulation-division (RND) superfamily, (e) the multidrug and toxic compound extrusion (MTCE) superfamily, and (f) the drug metabolite transporter (DMT) superfamily.

*P. aeruginosa* is widely prevalent in the hospital environment and usually causes colonization of the alimentary and respiratory tracts. A history of previous rectal colonization is typically present in most patients developing infections. Recent antibiotic therapy is a significant risk factor for rectal colonization by MDR* P. aeruginosa* in critically ill patients [39,59]. Intestinal colonization and previous use of antibiotics are key risk factors for *P. aeruginosa *infections. Pathogen-related factors that determine a worse outcome of* P. aeruginosa* infections include the presence of certain horizontally acquired genomic islands; infection by specific clonal lineages; and expression of virulence factors, such as elastase, type III secretion system (T3SS), and the production of cytotoxins [39]. Host factors such as age, immunosuppression, and underlying disease influence the outcome of *Pseudomonas *infections. Delayed adequate antimicrobial therapy is also independently associated with increased mortality. In one study, single-agent susceptibility rates for the 11,701 non-duplicate *P. aeruginosa* isolates ranged from 72.7% for fluoroquinolones and 85.0% for piperacillin-tazobactam [71]. Susceptibility rates were higher for blood isolates than for respiratory isolates [39]. The increasing prevalence of MDR or XDR *P. aeruginosa* isolates is associated with the spread of high-risk clones, such as ST175 [59].

*Acinetobacter spp*. is a gram-negative, non-fermenting coccobacillus strictly aerobic, oxidase-negative, catalase-positive, pleomorphic, and non-motile [41]. These bacteria are widespread in the environment in soil, water, and sewage. *Acinetobacter baumannii* causes opportunistic nosocomial infections involving patients on mechanical ventilation in intensive care units. *A. baumannii* can colonize new surfaces by the formation of a biofilm. Although polymyxin-resistant* A. baumannii* represents less than 1% of clinical isolates, its widespread dissemination, multidrug resistance, and multiple virulence factors make it a severe threat to public health worldwide [62]. It has been shown using molecular techniques that *A. baumannii *outbreaks have been primarily due to specific clones [41].* A. baumannii* commonly has extensive resistance to penicillins, cephalosporins, tetracyclines, macrolides, chloramphenicol, fluoroquinolones, aminoglycosides, and carbapenems. Polymyxins are the antibiotic of last resort to treat infections caused by XDR *A. baumannii*. Polymyxin acts by disrupting membrane integrity through the displacement of divalent cations in the outer membrane by binding to the lipopolysaccharide (LPS) and causing cell lysis. Unfortunately, lineages with low sensitivity to polymyxins have increased in Europe, Asia, and South America [63].

Polymyxin resistance in *A. baumannii* is attributed to changes in the outer membrane through phosphoethanolamine addition, loss of LPS, changes in osmoprotective amino acids, and overexpression of efflux pumps. Inactivation of the lipid A biosynthetic genes, *lpxA*,* lpxC*, or *lpxD*, results in a complete loss of surface LPS. Thus, the loss of LPS prevents the essential interaction between it and polymyxins [25,63]. Mutations identified in the *pmr *family of genes are also associated with colistin resistance. This family of genes encodes enzymes involved in the synthesis of lipid A, a component of LPS. Modification of lipid A protects the outer membrane from the binding and action of polymyxins. *A. baumannii *also possesses genes expressing efflux pumps related to antibiotic resistance, including resistance-nodulation division (RND), major facilitator (MF), multidrug-toxic compound extrusion (MATE), and small multidrug resistance (SMR) families. The *mcr*-encoding plasmid found initially in *E. coli *in China has been subsequently reported worldwide in other gram-negative bacteria, including *A. baumannii* [25].

Fungal Antimicrobial Resistance

The growing incidence of fungal infections in the hospital environment is alarming. Fungi are normal commensals on the human body but can cause invasive infections, particularly in immunocompromised patients. Risk factors for invasive fungal infection include the presence of a central venous catheter, invasive catheterization, diabetes mellitus, immunosuppression, receiving total parenteral nutrition, recent surgery, extended hospital stay, prolonged admission to the ICU, and having received broad-spectrum antibiotics [29,38]. Nosocomial outbreaks due to relatively uncommon fungal species such as *Exserohilum rostratum* and *Sarocladium kiliense* have occurred following the contamination of medical products [49]. However, significant MDR fungemia is usually caused by* Candida spp*., including *Candida glabrata, Candida parapsilosis*, and *Candida auris*.

*C. auris*, an MDR yeast species, is undoubtedly the most problematic species because of its ability to form a biofilm, colonize patients, and persist in the healthcare environment. First reported in 2009 in a Japanese patient, *C. auris* cases have since been reported, as of February 15, 2021, in 47 countries on all inhabited continents. *C. auris* isolates have been classified using whole-genome sequencing into four geographically distinct clonal populations [29]. *C. auris* is clinically significant because it has demonstrated resistance to multiple antifungal drugs, with some isolates resistant to all major antifungal classes (azoles, polyenes, and echinocandins). *C. auris* candidemia is associated with a 30-60% mortality rate. The transmissibility and extensive antifungal resistance characteristic of *C. auris *set it apart from other *Candida *species. In the United States, approximately 90% of isolates have been resistant to fluconazole, 30% to amphotericin B, and 5% to echinocandins compared to 10% of *C. glabrata* isolates exhibiting fluconazole resistance, and less than 10% exhibit echinocandin resistance [38].

In one study, 41% of patients received systemic antifungal therapy when *C. auris* was isolated. The median time from admission to infection was 19 days, 61% of patients had bloodstream infections, and 59% died. Interestingly, 41% of isolates were resistant to two antifungal classes, and 4% were resistant to three classes of antifungals [74]. In another study at a large tertiary hospital in China, the average detection rate was 0.29% over a decade. Non-*Candida albicans* was the main fungus, accounting for 62.5% of isolates. The drug resistance of non-*Candida albicans* was higher than that of *C. albicans*, among which *C. glabrata* had the highest resistance rate [38]. Molecular mechanisms underlying resistance in* Candida* species include* Erg11 *mutations, which mediate fluconazole resistance. Efflux pump activity also contributes to azole resistance. It is hypothesized that* FKS* mutations observed in *C. auris* isolates, such as the *S639F* mutation, are responsible for micafungin resistance. A mutation in a gene involved in ergosterol biosynthesis mediates resistance to amphotericin B via a reduction in ergosterol content in the fungal cell wall [29].

Biofilms

Biofilms are aggregates of microorganism communities that adhere irreversibly to abiotic or biotic surfaces through the production of extracellular polymeric material [40]. The self-produced polymeric matrix facilitates the formation of complex structures that promote antibiotic resistance through horizontal gene transfer and persister cells that result in chronic or recurring infections. Persister cells are dormant cells within biofilms that can tolerate high concentrations of antibiotic agents [45]. Biofilms play an essential role in healthcare-associated bacterial and fungal infections. They are more resistant to antimicrobials due to their (a) physiological state, (b) cell density, (c) quorum sensing abilities, (d) protective extracellular matrix, (e) upregulation of drug efflux pumps, (f) increased expression of resistance genes, and (g) presence of persister cells. The significance of the drug efflux pump mechanism in biofilms was observed in a study involving *C. albicans* that showed that strains lacking* Cdr1p, Cdr2p*, and *Mdr1p* pumps were more susceptible to fluconazole at the initial stages of biofilm formation compared to the wildtype. Again, the expression of the efflux pump, *AfuMDR4*, was notably upregulated in vivo upon exposure to voriconazole [45].

Treatment options

Policy

The use of an antibiotic policy fosters improved prescribing practices and evidence-based antimicrobial use. An antimicrobial stewardship program involves a multifaceted and multidisciplinary approach to achieving the following goals: (a) controlling antimicrobial resistance, (b) improving clinical outcomes, and (c) reducing costs by improving antimicrobial use [56]. The core components of an antibiotic policy must include antimicrobial stewardship, especially the development of prescribing guidelines and standards of care, as well as infection prevention strategies such as hand hygiene, hospital cleaning, and disinfection. Active surveillance is required in outbreaks of MDR, XDR, and PDR infections. Antibiotic resistance surveillance and comparisons of prescribing practices are beneficial feedback activities once effectively communicated to healthcare practitioners [23].

One RCT identified high-certainty evidence that interventions in antimicrobial stewardship programs enabled physicians to improve their antibiotic prescribing practices, reduced the length of stay in hospitals by 1.12 days, and did not increase mortality [12]. Interventions were categorized into restrictive techniques, which incorporate policies to make physicians prescribe properly, and enablement techniques, which provide feedback and advice to help physicians prescribe properly. Enablement interventions were more effective in improving prescribing practices [12]. The best current intervention for optimizing antibiotic use is to have clear guidelines for using an antibiotic regimen. The antibiotic regimen selected should have the highest efficacy for a confirmed infection. The benefits of such intervention include improved clinical cure rates, less antibiotic toxicity, fewer *Clostrididoides difficile* infections, less disruption of the gut microbiome, and fewer MDR infections. The overarching goal is to provide timely, appropriate antibiotics while avoiding antimicrobial resistance. Timely initial appropriate antibiotics are a critical determining factor of outcomes in severe infections. Several studies have demonstrated that inappropriate initial antimicrobial therapy was independently associated with increased mortality and extended hospital stay [12,56].

Appropriate initial antimicrobial therapy can be achieved using a local antibiogram or rapid molecular identification methods. Keeping a local antibiogram is imperative to guide and periodically review antimicrobial stewardship programs. An antibiogram records the overall profile of antimicrobial susceptibility testing results of specific microbes to a battery of antimicrobial drugs. It helps to guide empiric treatment while microbiology culture and sensitivity results are pending. Additionally, they can be used to detect, monitor, and investigate trends in antimicrobial resistance. Rapid microbiological identification methods, such as PCR, are currently in clinical use to identify resistance genes and quickly guide initial targeted narrow-spectrum antibiotic treatment until final microbiological culture and sensitivity results are known. The ability to determine susceptibility patterns in hours rather than in days is handy to the clinician, especially in severely ill patients or those with bacteremia. The drawback is that these methods do not differentiate colonization from infection.

Non-antibiotic Measures

The healthy microbiota provides protective functions, including preventing colonization and infection via competitive pressure. Antibiotic exposure is associated with disrupting the microbiota that selects for resistance in the gut microbiome. Novel methods to exploit protective mechanisms provided by intact microbiota may provide the key to preventing the spread of MDR organisms in the healthcare setting [55,66]. It is hypothesized that probiotics may effectively decolonize and prevent MDR infections by promoting healthy intestinal microbiota. Some evidence-based analyses from various human studies and animal models have shown the clinical potential of probiotics against infectious diseases, diarrhea, intestinal infections, inflammatory diseases, and antibiotic-associated diarrhea. These studies suggest that it is possible to counteract microbial colonization and antimicrobial resistance spread [42]. However, this is yet to be proven by an RCT. In several RCTs, probiotic drugs were ineffective for decolonizing hospitalized patients harboring MDR gram-negative bacilli and preventing subsequent infections. They did not reduce the in-hospital length of stay, the incidence of adverse events, and in-hospital mortality rates [11].

Antibiotic Measures

Decolonization is a strategy to reduce the incidence of healthcare-associated infections. Decolonization involves the use of topical antimicrobial agents to reduce the bacterial burden on specific sites of the human body, including the nares and the skin. There have been only a few multicenter, randomized trials evaluating decolonization. Of the few that exist, even fewer have compared decolonizing agents head-to-head to determine the superiority of an agent or a decolonizing protocol [50]. The most robust evidence for decolonization is to prevent surgical site infections among surgical patients. The populations that benefit the most from decolonization are cardiac and orthopedic surgery patients. The common agents used for decolonization include chlorhexidine, mupirocin, and povidone-iodine [18,50]. Mupirocin is used for nasal decolonization for methicillin-resistant *Staphylococcus aureus* (MRSA). Chlorhexidine gluconate (CHG) is the decolonization agent with the most substantial evidence base for oral and skin cleansing. A meta-analysis revealed that 2% chlorhexidine bathing significantly reduced hospital-acquired infection incidence and MDR organisms in ICUs [18].

The reducing sensitivity of MRSA, coagulase-negative *Staphylococcus spp.*, and *Enterococcus species* to vancomycin is a worrisome threat [36]. The minimum inhibitory concentration (MIC) creep phenomenon is a notable cause of increasing resistance to vancomycin often occurring because of underdosing and excessive use. MIC creep refers to the gradual but steady increase in the levels of MIC standards for MRSA isolates. This results in poor clinical response, high relapse rates, and treatment failures. The two leading alternatives for vancomycin-resistant enterococci (VRE) and VRSA treatment are linezolid and daptomycin, with clinical success rates of 50-80% as a first-line drug and 50-59% as salvage therapy for VRE bacteremia, respectively [75].Enterococci spp. commonly have intrinsic resistance to penicillin monotherapy. However, susceptibility increases when antibiotics with activity against the bacterial cell wall, such as β-lactams, are used synergistically. Double β-lactam therapy is effective in enterococci endocarditis, although no studies have shown efficacy for such therapy in other sites such as deep-seated abscesses and osteomyelitis. Again, ampicillin, an aminopenicillin, is remarkably effective against enterococci infections when used in synergy with gentamicin, an aminoglycoside. Nephrotoxicity commonly limits the use of gentamicin.

For gram-negative bacteria, ceftolozane/tazobactam is especially active against *P. aeruginosa* (from the intrinsic activity of ceftolozane, a semi-synthetic fifth-generation cephalosporin). In contrast, the addition of tazobactam confers activity against most ESBL)producers. It is approved to treat complicated urinary tract infections, intra-abdominal infections, and nosocomial pneumonia. Avibactam is a novel β-lactamase inhibitor that inactivates class A [including *K. pneumoniae* carbapenemase (*KPC*)], class C (*AmpC*), and some class D (*OXA*) β-lactamases. The combination of ceftazidime/avibactam inhibited 82% and 76% of MDR and XDR strains, respectively. The susceptibility of *P. aeruginosa* toward ceftazidime increases from 65% to 94% when used in combination with avibactam [59]. Another novel β-lactamase, relebactam, inhibits Ambler class A and class C cephalosporinases, effectively boosting imipenem activity against resistant *K. pneumoniae* carbapenemase (KPC) and *P. aeruginosa* [77]. The combination of imipenem, cilastatin, and relebactam is approved for the treatment of complicated intra-abdominal infections and complicated urinary tract infections.

Newer antibiotics discovered in the last decade include cefiderocol, plazomicin, and eravacycline. Cefiderocol is a novel siderophore cephalosporin that binds to ferric iron which is required for bacterial growth and virulence. Cefiderocol is actively transported across the outer membrane resulting in high concentrations in the periplasmic space, where it exerts a bactericidal effect by binding to penicillin-binding proteins and inhibiting cell wall synthesis. It has been shown to have potent in-vitro activity against MDR gram-negative bacteria including *Enterobacterales *(>90% of isolates),* P. aeruginosa*, *A. baumannii, *and *Stenotrophomonas maltophilia* [78]. However, cefiderocol has a label warning for higher all-cause mortality versus other antibiotics in critically ill patients with MDR gram-negative bacteria with a mortality rate of 34% for cefiderocol vs. 18% in the best-available therapy group [78]. Plazomicin is a synthetic aminoglycoside approved by the U.S. Food and Drug Administration (FDA) for complicated urinary tract infections active against >95% of *Enterobacterales* isolates. It is active against ESBL isolates and against 84.6% to 97.6% of carbapenem-resistant isolates. The presence of aminoglycoside-modifying enzymes does not inactivate plazomicin, and it is active against 52.2% of isolates that are resistant to three members of the aminoglycoside drug class [79]. Eravacycline is a fluorocycline of the tetracycline class. The FDA has approved eravacycline for the treatment of complicated intrabadominal infections. Eravacycline is active against ESBL* E. coli* and *K. pneumoniae*. It has activity against* A. baumannii *andcarbapenem-resistant *Enterobacterales* but has limited activity against *P. aeruginosa*. Eravacycline has been also investigated for complicated urinary tract infections but showed lower cure rates (84.8% vs. 94.8%) and (60.4% vs. 66.9%) than ertapenem and levofloxacin, respectively [79].

The overall incidence of non-ventilator hospital-acquired pneumonia was 1.6%, representing a rate of 3.63 per 1,000 patient days in the United States [34]. In one study, ceftazidime/avibactam was non-inferior to meropenem to treat healthcare-associated pneumonia/ventilator-associated bacterial pneumonia [53]. It is a valid option against carbapenem-resistant *Enterobacteriaceae* (CRE). Meropenem/vaborbactam, another novel therapeutic option, displayed a non-significant trend toward lower mortality in patients with CRE infections and penetrated well into the lung [53]. Vaborbactam inhibits class A and C β-lactamases but not class B or D lactamases. In one study, there was no statistical difference in all-cause mortality between monotherapy and combination therapy to treat people with ventilator-associated pneumonia (VAP) [10]. In VAP, a short treatment course of about seven days is validated, even though a longer treatment course may still be recommended for patients with a slower clinical response. Usually, carbapenem monotherapy is used for VAP, including MDR strains. However, there was no statistical difference in all-cause mortality between carbapenem and non-carbapenem therapies. However, carbapenems are associated with a statistically significant increase in clinical cures [10]. On the other hand, a meta-analysis identified significantly higher superinfection with imipenem than non-carbapenems. Superinfection is a new microbial infection occurring after or in addition to an earlier infection usually following treatment with broad-spectrum antibiotics. Superinfection was statistically higher when carbapenems were used compared to other antipseudomonal beta-lactams [20].

Drug concentrations in the airways can be 100-fold higher when antibiotics are administered through the aerosol route in mechanically ventilated patients. Several studies demonstrate a reduction of bacterial load and a good safety profile with aerosolized colistin, or aminoglycosides compared to the intravenous route [8,25]. Accordingly, aerosolized antibiotics are now increasingly used, especially in gram-negative VAP and, more specifically, with MDR strains. However, one rational approach for XDR strains, especially *A. Baumannii* is to consider combining colistin and carbapenem therapy, particularly when carbapenem MICs are elevated [25]. This approach attempts to supplement the therapeutic effect of the last-line polymyxin therapy with systemic therapy with a carbapenem or another agent.

In VAP caused by MDR *A. baumannii*, treatment with intravenous meropenem, colistin, and nebulized tobramycin was just as effective as intravenous meropenem, injectable colistin, and nebulized colistin, with no significant difference between clinical pulmonary infection score and creatinine level in both groups, suggesting that nebulized tobramycin is non-inferior to nebulized colistin [8]. Finally, in cases of non-bacteremic XDR *Acinetobacter* *spp.* pneumonia, the addition of inhaled colistin minimizes toxicity and maximizes levels delivered to the lung.

Treatment of Fungal Infections

*C. auris* infections pose a real treatment challenge due to the formation of biofilms and resistance mechanisms. Echinocandins are the recommended first-line treatment in adults. An alternative is liposomal amphotericin B. Antifungal susceptibility testing is required to inform targeted treatment. Outbreaks of *Candida* require hypervigilance, rapid diagnostic methods, and new molecular typing tools such as whole-genome sequencing (WGS), prompt investigation, and aggressive interventions, including notification of public health agencies [38,49]. For a suspected *C. auris *infection, the Centers for Disease Control and Prevention (CDC) recommends the identification of species from non-sterile sites when there is an invasive disease, colonization, or infection is detected in a unit or facility, or when a patient has had an overnight stay within the previous year in a healthcare facility in a country with documented *C. auris* transmission [29].

Alternative Treatments

Antimicrobial lock therapy (ALT) is an alternative therapy for biofilm-related infections associated with medical devices such as central vein catheters. ALT involves instilling antimicrobial agents, which exceed the MIC by 100- to 1,000-fold within an intravascular catheter lumen. The antibiotic will stay locked over a specific time, usually 24 hours for most agents [40]. Its efficacy is disputed, given the lack of source control. However, ALT is used when central catheters cannot be removed for clinical reasons. Several clinical studies indicate that ALT is an effective method for preventing *Candida *colonization without removing catheters but is yet to be confirmed by a large RCT [40]. In cases of *Candida* fungemia, however, removal of the catheter remains the standard of care.

Future directions

Antimicrobial coatings are promising options to eradicate biofilm-related infections. Medical devices typically associated with biofilm formation are coated with anti-biofilm layers to prevent the adherence of microbes. These coated surfaces serve as contact-killing surfaces preventing the formation of biofilms, as observed in cases of central line-associated bloodstream infections, catheter-associated urinary tract infections, and VAPs. Nanotechnology is a complementary therapeutic agent that employs quantum-dot, carbon nanotubes, and carbon-based nanoparticles (NPs) to disrupt biofilms and deliver antimicrobials directly to the targeted cells or pathogens without drug degradation. NPs are considered promising alternatives to antibiotics and effective against gram-positive and gram-negative bacteria. Natural NPs, polymer-based nanomaterials, and metallic NPs are cost-effective and may be exploited as antimicrobial coatings on the surface of medical devices for various biomedical applications [45].

Antimicrobial photodynamic therapy (aPDT), or photodynamic activation (PDI), is an alternative treatment modality for localized biofilm infections. Its mechanism of action results from synergism between non-toxic photosensitizer dye, molecular oxygen, and visible light. The principle behind aPDT is that exposure to a light source at a specific wavelength triggers the photosensitizer dyes to generate sufficient reactive oxygen species from molecular oxygen that cause damage and microbial cell death without exerting toxic effects in the host. These modalities are relatively low cost, and widespread adoption will further increase their cost-effectiveness.

Limitations

Although this review is based on a systematic analysis of the medical literature, it reflects the inherent biases of the writing team. We have summarized the most compelling areas of current investigations based on the literature and the experience of the writing committee. As new research becomes available, additions to the priorities of this research agenda should be considered.

## Conclusions

Clinicians and scientists have long realized the remarkable ability of microorganisms to survive via evolving resistance to antibiotics. Infections caused by MDR/XDR strains are a cause of concern as they compromise the selection of appropriate empiric and definitive antimicrobial treatments. The knowledge about the variety of molecular mechanisms of antimicrobial resistance has expanded tremendously via advances in genomics and proteomics. Many molecular mechanisms that promote resistance have been elucidated; however, novel antibiotic drug development has not kept pace in tandem. The judicious use of antibiotics through antimicrobial stewardship, good clinical practice, and good public health practices are imperative to stem the tide of increasing drug resistance. There is a need for fundamental studies to answer questions regarding the development and use of new antibiotics and novel strategies for treating and preventing MDR/XDR/PDR bacterial infections.
